# Optic nerve changes in chronic sinusitis patients: Correlation with disease severity and relevant sinus location

**DOI:** 10.1371/journal.pone.0199875

**Published:** 2018-07-10

**Authors:** Young Hyo Kim, Joseph Kim, Min Gu Kang, Dae Hyung Lee, Hee Seung Chin, Tae Young Jang, Na Rae Kim

**Affiliations:** 1 Department of Otorhinolaryngology-Head and Neck Surgery, Inha University School of Medicine, Incheon, Korea; 2 Department of Ophthalmology and Inha Vision Science Laboratory, Inha University School of Medicine, Incheon, Korea; 3 Clinical Trial Center, Inha University Hospital, Incheon, Korea; University of Botswana Faculty of Medicine, BOTSWANA

## Abstract

**Purpose:**

This study was to evaluate whether optic nerve damage occurs in eyes with adjacent chronic sinusitis.

**Methods:**

Data were collected from eighty-eight eyes of 46 chronic sinusitis patients and 93 eyes of 57 normal controls. Visual sensitivity using standard automated perimetry (SAP) and inner retinal thickness using optical coherence tomography (OCT) were measured. The Lund-Mackay system was used to quantify radiographic findings on the ostiomeatal unit CT scan with a numerical score representing the severity of sinusitis.

**Results:**

There was a significant positive correlation between the pattern standard deviation (dB) and Lund-Mackay score (P = 0.031). Nasal retinal nerve fiber layer (RNFL) thickness, average, minimum, superotemporal, superior, superonasal, and inferonasal ganglion cell-inner plexiform layer (GCIPL) thickness were negatively correlated significantly with Lund-Mackay score (all, P < 0.05). Eyes with grade 2 opacification of the posterior ethmoid sinus showed a significantly lower mean deviation (dB) and higher pattern standard deviation (dB) than those with clear respective sinuses (P = 0.007 and <0.001, respectively). Eyes with grades 1,2 and 3 opacification of the sphenoid sinus had a significantly less average RNFL thickness (P = 0.004, <0.001, and <0.001, respectively) and a significantly less average GCIPL thickness (P = 0.004, 0.003, and 0.003, respectively) than those with a clear sphenoid sinus.

**Conclusions:**

Structural and functional optic nerve changes were correlated with the severity of chronic sinusitis. Inflammation of the posterior ethmoid and sphenoid sinuses was associated with optic nerve changes to a greater extent than that of the other paranasal sinuses.

## Introduction

Sinusitis, defined as inflammation of one or more of the paranasal sinuses, is characterized as acute when lasting less than 4 weeks, subacute when lasting 4 to 12 weeks, and chronic when lasting longer than 12 weeks.[1] Chronic sinusitis is a common and often debilitating disease affecting more than 30 million Americans.[2] The recent prevalence of chronic sinusitis (with or without polyps) has been reported to be as high as 8.4% in the Korean population.[3]

The optic nerve or II cranial nerve is not a true cranial nerve but a fiber tract of the brain formed by axons of the retinal ganglion cells that become myelinated by oligodendrocytes as they leave the optic disc.[4] The optic nerve can be divided into four segments: intraocular, intraorbital, intracanalicular and intracranial.[5, 6] Anatomic studies have documented the relationship between the optic nerve and the paranasal sinuses. An impression of the optic nerve in the superolateral wall of the sphenoid sinus is frequently seen and reflects their intimate relationship.[7] Even bony dehiscence of the sphenoid sinus directly over the optic nerve has also been found in 4% of cadavers.[8, 9] There have been sporadic reports of optic neuropathy caused by a mechanical compression of the optic nerve, circulatory disturbance of the vasa nervorum due to mechanical compression, and optic neuritis due to inflammation, such as polyps in the Onodi cell,[10] invasive sino-orbital aspergillosis,[11] acute bacterial sphenoid sinusitis,[12] eosinophilic mucin rhinosinusitis,[13] allergic fungal sinusitis,[14] and sinusitis adjacent to optic nerve.[15]

Here, the authors studied whether optic nerve change occurred in the eyes of those with an adjacent chronic sinusitis by measuring the inner retinal thickness using optical coherence tomography (OCT) and by measuring visual sensitivity using standard automated perimetry (SAP). The authors also investigated whether the severity of sinusitis and the location of the relevant sinus were related to the extent of optic nerve damage.

## Materials and methods

In this prospective study conducted over a 5-year period (March 2011 to February 2016) in a tertiary eye care center and department of otorhinolaryngology (Inha University Hospital, Incheon, Korea), consecutive subjects (58 chronic sinusitis patients and 57 normal controls) were recruited. This study received approval from the institutional review board of Inha University Hospital (IUH-IRB 13–0480) and was conducted in accordance with the Declaration of Helsinki. Patients agreed and participated in this study by their own free will.

### Patients and control groups

Patients with chronic sinusitis, who had symptoms for at least 12 weeks and whose symptoms persisted despite adequate medication, were enrolled. The average duration of symptoms of sinusitis obtained from the patient at the time of ENT examination was 2.09 ± 3.39 years. Their chronic sinusitis was confirmed by endoscopy (mucopurulent nasal discharge and/or nasal polyposis) and computed tomography. On the other hand, those with negative endoscopic and CT findings for sinusitis and without symptoms of chronic sinusitis were enrolled as controls. We excluded patients with an unstable systemic disease and pregnant or lactating women.

### Ophthalmologic examinations

The ophthalmologic examination was performed within one month before and after the ENT examination. The mean interval between ophthalmologic examination and ENT was 21.18 ± 32.05 days. Ophthalmologic eligibility criteria were determined based on a complete ophthalmologic examination, which included a review of the patient’s medical history, best-corrected visual acuity measurements through manifest refraction, Goldmann applanation tonometry, slit-lamp examination of the anterior segment, gonioscopy, dilated fundus examination, red-free fundus photography (Canon, Tokyo, Japan), Humphrey standard automated perimetry visual test (Carl Zeiss Meditec), and Cirrus OCT (Carl Zeiss Meditec). Eyes were recruited from subjects with no history or evidence of intraocular surgery, no media opacity on slit-lamp examination, no history of glaucoma (personally or in a first-degree relative), no smoking and alcoholic habits, no family history of optic neuropathy, and no retinal pathologic features.

A VF test was performed by automated static perimetry (Humphrey Field analyzer with the Swedish Interactive Thresholding Algorithm [SITA] standard 24–2 test program; Carl Zeiss Meditec, Dublin, CA). The results of VF testing were considered reliable when fixation loss was < 20%, and false-positive and false-negative errors were both < 15%.

In all participants, a Cirrus HD-OCT (Carl Zeiss Meditec, software version 6.0) was used to acquire one optic disc cube protocol and one macular cube protocol in each qualifying eye. The optic disc cube protocol was designed to position the cube scan on the optic nerve head (ONH). This protocol generated a cube of data through a 6-mm-square grid by acquiring a series of 200 horizontal scan lines, each composed of 200 A-scans (40,000 points). The retinal nerve fiber layer (RNFL) thickness at each pixel was measured and an RNFL thickness map was generated. A calculation circle 3.46 mm in diameter and consisting of 256 A-scans was automatically positioned around the optic disc and the mean and sectoral (temporal, superior, nasal, and inferior) RNFL thicknesses were subsequently measured.

Macular cube scans in Cirrus OCT analyze a 6x6 mm^2^ area of the fovea using a macular cube 512x128 or 200x200 scan protocol. The Ganglion Cell Analysis algorithm identifies the outer boundary of the RNFL and the outer boundary of the inner plexiform layer. The difference between the RNFL and the IPL outer boundary segmentations yields the combined thickness of the retinal ganglion cell layer and the inner plexiform layer, which in turn provides a measurement of macular ganglion cell-inner plexiform layer (GCIPL) thickness within a 14.13-mm^2^ elliptical annulus area centered on the fovea. The mean, minimum, and six individual sectors (superior, superonasal, inferonasal, inferior, inferotemporal, and superotemporal) of GCIPL thickness were determined.

**Fig 1:**
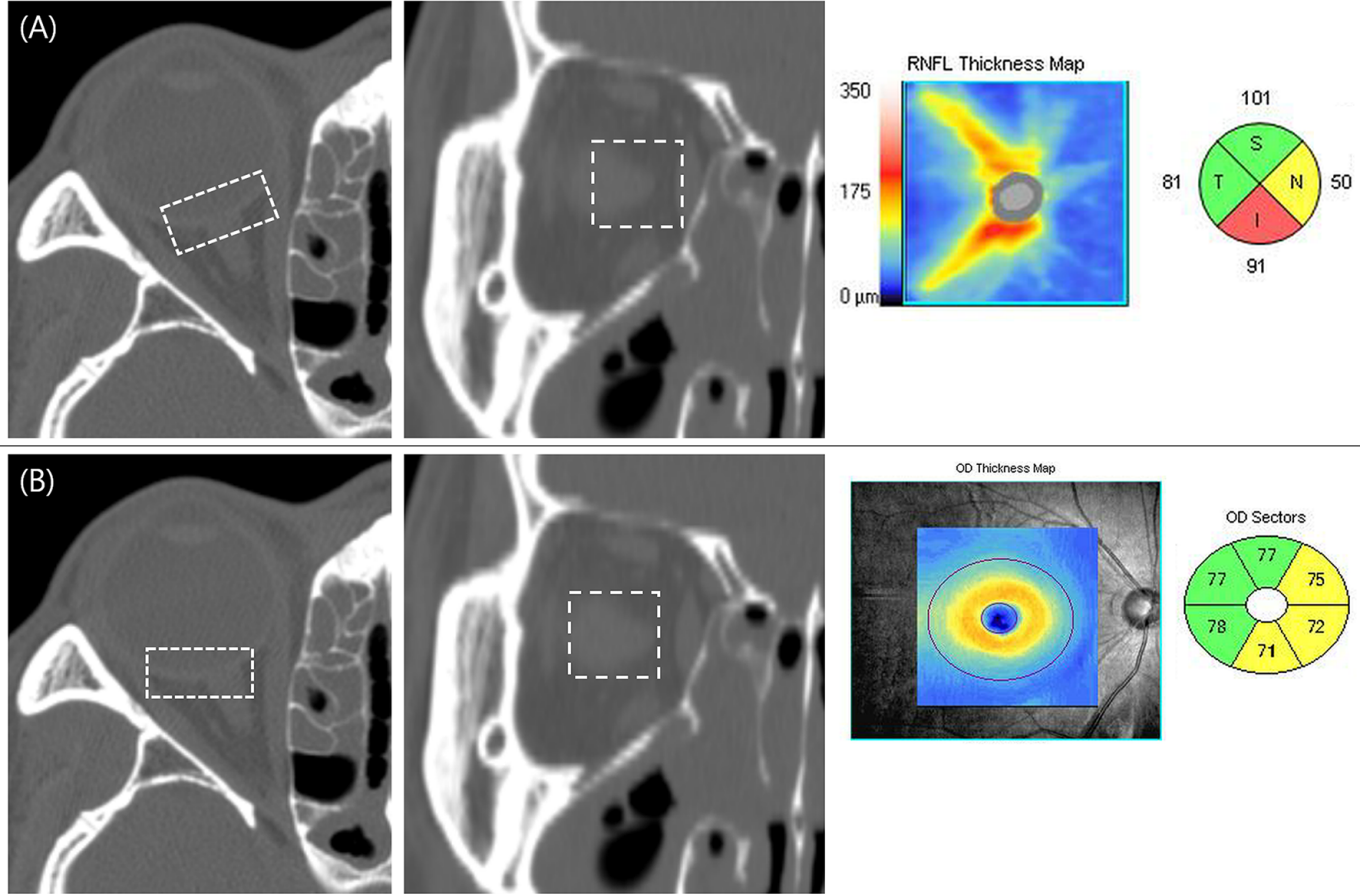
Laser interferometry scan in chronic sinusitis patients. (A) The location of the peripapillary scan with regards to the sagittal and coronal section of the ostiomeatal unit CT. OCT maps showing the retinal nerve fiber layer thickness (T = temporal, S = superior, N = nasal, and I = inferior). (B) The location of the macular scan with regards to the ostiomeatal unit CT. OCT maps showing the ganglion cell-inner plexiform layer thickness.

The location of the OCT scan with respect to the sagittal and coronal sections of the ostiomeatal unit CT is presented in Fig 1.

### Lund-Mackay CT scoring

Ostiomeatal unit CT scans of the paranasal sinuses were acquired for all patients. The Lund-Mackay system was used to quantify the radiographic findings on the sinus CT scan with a numerical score. The right or left sinuses were respectively divided into six portions, including the maxillary sinus, anterior ethmoid sinuses, posterior ethmoid sinuses, sphenoid sinus, frontal sinus, and ostiomeatal complex. The severity of sinus mucosal inflammation or fluid accumulation was scored as 0 (no opacification), 1 (1–49% opacification), 2 (50–99% opacification), or 3 (total opacification). Undeveloped sinuses were not scored. In addition, the ostiomeatal complex was scored as either 0 (patent) or 2 (occluded) because it is difficult to describe the ostiomeatal complex with any gradation.

In this study, unilateral Lund-Mackay scoring was used to assess each eye separately. The five scores for the various unilateral sinuses from either the left or the right and one ipsilateral ostiomeatal complex were summed to give a separate unilateral total Lund-Mackay score that could range from 0 (complete lucency of all sinuses) to 17 (complete opacity of all sinuses).

### Classification according to the relationship between the paranasal sinuses and optic nerve

The patients who had a sphenoid sinus and/or posterior ethmoid sinus involvement on CT scan were categorized into four groups based on the classification by Delano,[16] which associates the relationship between the sphenoid sinus, posterior ethmoid sinus, and the optic nerve. The optic nerve was categorized as (1) Type 1 when it was found to be lying adjacent to the superior and lateral walls of the sphenoid sinus, (2) Type 2 when it was found to make an indentation on the sphenoid sinus, (3) Type 3 when it traversed the sphenoid sinus, and (4) Type 4 when it was adjacent to the sphenoid and posterior ethmoid sinuses and covered by aerated cells.[16]

### Statistics

When both eyes of a patient were eligible, both were included in the analysis. Visual sensitivity parameters and retinal thickness parameters were compared between the chronic sinusitis group and normal control group using the generalized estimating equation (GEE) adjusted for inter-eye correlation. GEE adjusted for age, sex, spherical equivalent, and inter-eye correlation was used to assess the relationship between the unilateral total Lund-Mackay score and intraocular pressure, visual field sensitivity parameters, and OCT retinal thickness parameters in the ipsilateral eye. We investigated whether chronic sinusitis in specific paranasal sinuses were related to the ophthalmic parameters in the ipsilateral eye using GEE adjusted for age, sex, spherical equivalent, inter-eye correlation, and other sinus opacification. Ophthalmic features among the four groups that were classified by the anatomic relationship between the paranasal sinuses and optic nerve were compared using GEE adjusted for age, sex, spherical equivalent, and inter-eye correlation, and the comparisons were done by post-hoc analysis. Data analysis was carried out using the Statistical Package for Social Sciences (SPSS) version 19.0 (SPSS, Inc., Chicago, IL, USA).

## Results

There were 88 eyes of 46 patients in the chronic sinusitis group and 93 eyes of 57 subjects in the normal control group. The time difference between OMU CT study and the visual field and OCT was 49.71 ± 28.51 days. The mean age of the chronic sinusitis and normal control group was 47.13 ± 12.53 and 50.93 ± 13.51 years (mean ± SD) (P = 0.143) (Table 1).

**Table 1:** Baseline characteristics of study population.

	Control (n = 57)	Sinusitis (n = 46)	P
Age (y)	50.93 ± 13.51	47.13 ± 12.53	0.143
M: F	33: 24	35:11	0.053
DM (Yes)	2 (4.2%)	7 (15.6%)	0.084*
Hypertension (Yes)	7 (14.6%)	6 (13.3%)	0.862

DM = diabetes mellitus.

Data are presented as the mean ± SE.

Chi-square test

*Fisher’s exact test

The mean intraocular pressure (IOP) for patients with chronic sinusitis and normal controls was 14.26 ± 0.53 and 15.03 ± 0.48 (mean ± SE), respectively (P = 0.283). The corredted visual acuity for patients with chronic sinusitis and normal controls was 0.08 ± 0.14 and 0.09 ± 0.16 (LogMAR), respectivlely (P = 0.651). The visual field mean deviation of the chronic sinusitis and normal control groups were -1.44 ± 0.29 dB and -0.64 ± 0.27 (mean ± SE) (P = 0.046). The average and sectoral RNFL and the average and sectoral GCIPL thickness measured using OCT did not differ significantly between those with chronic sinusitis and the normal controls (Table 2).

**Table 2:** Ophthalmic characteristics according to the presence or absence of chronic sinusitis.

	Control eyes (n = 93)	Sinusitis eyes (n = 88)	P
Intraocular pressure (mmHg)	15.03 ± 0.48	14.26 ± 0.53	0.283
Spherical equivalent (Diopter)	-1.00 ± 0.32	-0.57 ± 0.24	0.290
Corrected visual acuity (LogMAR)	0.09 ± 0.16	0.08 ± 0.14	0.651
VF			
Mean deviation	-0.64 ± 0.27	-1.44 ± 0.29	**0.046**
Pattern standard deviation	1.68 ± 0.15	1.88 ± 0.15	0.337
OCT RNFL parameter (μm)			
Average thickness	96.60 ± 1.19	95.93 ± 1.61	0.735
Temporal thickness	70.72 ±1.58	69.38 ± 1.49	0.538
Superior thickness	122.40 ± 1.80	120.40 ± 2.42	0.510
Nasal thickness	69.77 ± 1.24	70.77 ± 1.42	0.592
Inferior thickness	123.44 ± 1.96	124.17 ± 2.87	0.835
OCT GCIPL parameter (μm)			
Average thickness	82.80 ± 0.78	83.05 ± 0.99	0.841
Minimum thickness	79.77 ± 0.89	80.43 ± 1.03	0.631
Superotemporal thickness	82.28 ± 0.80	82.27 ± 0.93	0.995
Superior thickness	83.35 ± 0.84	84.00 ± 1.06	0.631
Superonasal thickness	84.76 ± 0.87	85.37 ± 1.10	0.663
Inferonasal thickness	82.45 ± 0.83	83.19 ± 1.05	0.583
Inferior thickness	80.40 ± 0.84	80.68 ± 0.95	0.826
Inferotemporal thickness	83.31 ± 0.78	83.73 ± 0.95	0.732

GCIPL = ganglion cell-inner plexiform layer; OCT = Optical coherence tomography; RNFL = retinal nerve fiber layer; VF = visual field.

Data are presented as the mean ± SE.

Analyzed by generalized estimating equation adjusting inter-eye correlation.

Analysis showing the relationship between the unilateral total Lund-Mackay score and ipsilateral ophthalmic parameters is presented in Table 3.

**Table 3:** Relationship of unilateral total Lund-Mackay score and ipsilateral ophthalmic parameters in patients with chronic sinusitis.

	Lund-Mackay score		
	beta	SE	P
Intraocular pressure	0.124	0.094	0.190
VF			
Mean deviation	-0.045	0.088	0.613
Pattern standard deviation	0.096	0.044	**0.031**
OCT RNFL parameters			
Average thickness	-0.298	0.326	0.360
Temporal thickness	0.096	0.300	0.749
Superior thickness	-0.073	0.442	0.869
Nasal thickness	**-0.821**	**0.289**	**0.004**
Inferior thickness	-0.315	0.625	0.614
OCT GCIPL parameter (μm)			
Average thickness	-0.445	0.163	**0.006**
Minimum thickness	-0.367	0.173	**0.034**
Superotemporal thickness	-0.411	0.143	**0.004**
Superior thickness	-0.500	0.168	**0.003**
Superonasal thickness	-0.643	0.179	**<0.001**
Inferonasal thickness	-0.581	0.217	**0.008**
Inferior thickness	-0.248	0.193	0.198
Inferotemporal thickness	-0.163	0.190	0.390

GCIPL = ganglion cell-inner plexiform layer; OCT = Optical coherence tomography; RNFL = retinal nerve fiber layer; VF = visual field.

**Fig 2:**
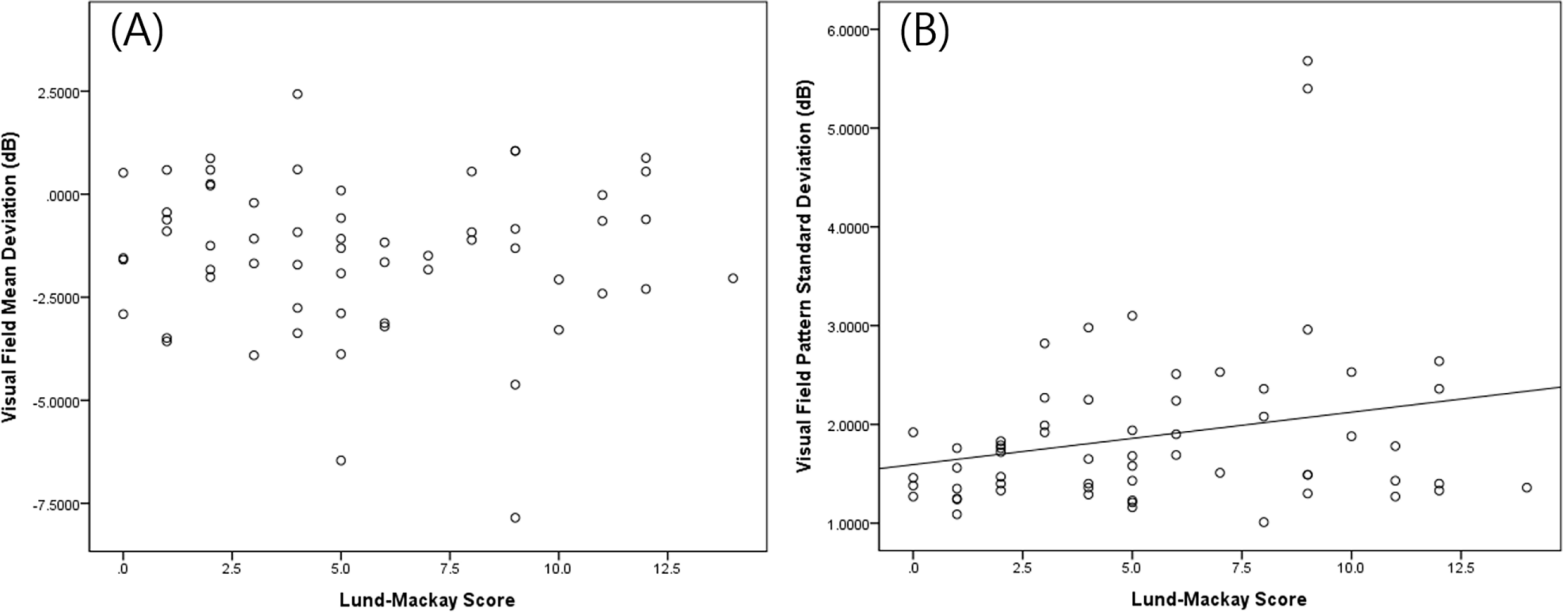
Multivariable-adjusted relationship between the Lund-Mackay score and ophthalmic functional parameters. (A) Visual field mean deviation (beta = -0.027, SE = 0.052, P = 0.607). (B) Visual field pattern standard deviation (beta = 0.042, SE = 0.024, P = 0.083).

With regard to the visual sensitivity parameters, the unilateral total Lund-Mackay score showed a significant positive correlation with the pattern standard deviation (beta = 0.096, P = 0.031) and no significant correlation with the mean deviation (beta = -0.045, P = 0.613) (Fig 2).

**Fig 3:**
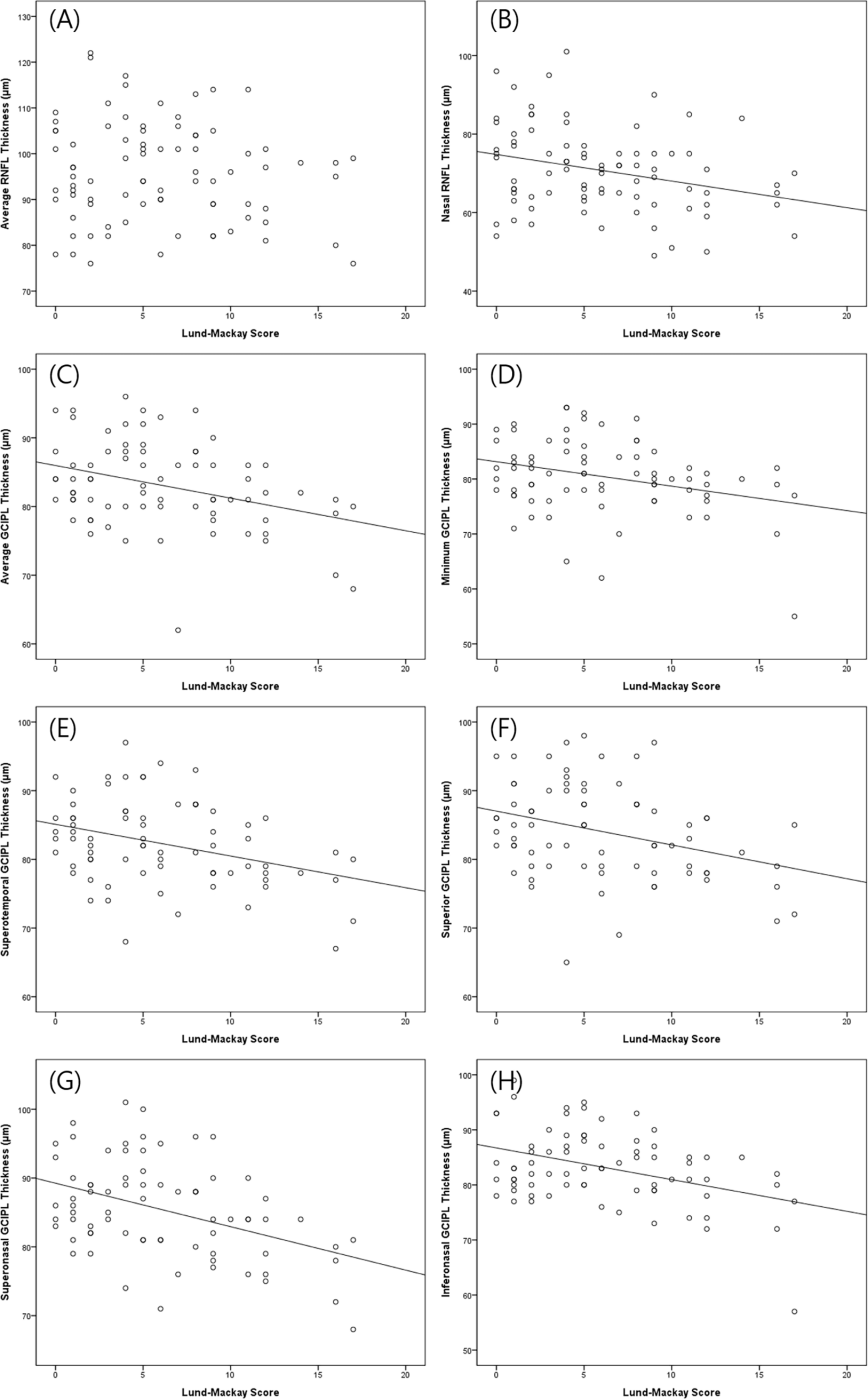
Multivariable-adjusted relationship between the Lund-Mackay score and ophthalmic structural parameters. (A) Average retinal nerve fiber layer (RNFL) thickness obtained by optical coherence tomography. (B) Nasal RNFL thickness. (C) Average ganglion cell-inner plexiform layer (GCIPL) thickness. (D) Minimum GCIPL thickness. (E) Superotemporal GCIPL thickness. (F) Superior GCIPL thickness. (G) Superonasal GCIPL thickness. (H) Inferonasal GCIPL thickness.

In the analysis between the Lund-Mackay score and OCT RNFL parameter, the average, temporal, superior, and inferior RNFL thickness did not correlate with the Lund-Mackay score. However, there was significant correlation between the Lund-Mackay score and nasal RNFL thickness; a higher Lund-Mackay score was associated with a thinner nasal RNFL thickness (beta = -0.821, P = 0.004). In the analysis between the Lund-Mackay score and OCT GCIPL parameter, the average, minimum, superotemporal, superior, superonasal, and inferonasal GCIPL thickness were negatively correlated with the score (all P < 0.05) (Fig 3).

The relationship between the functional and structural parameters with opacification of specific sinuses was investigated. In this analysis, the eyes with ipsilateral posterior ethmoid sinus opacification had a lower visual sensitivity; eyes with grade 2 opacification in the adjacent posterior ethmoid sinus had a lower mean deviation and higher pattern standard deviation compared to eyes with clear posterior ethmoid sinuses (beta = -2.96 and 1.69, P = 0.007 and <0.001, respectively). Eyes with grade 1 opacification (1–49% opacification) of an adjacent sphenoid sinus showed thinner RNFL (beta = -12.87) compared to eyes with a clear adjacent sphenoid sinus (P = 0.004). Sphenoid sinuses with grade 2 (50–99% opacification) and grade 3 (total opacification) opacification were related to a thinner RNFL of ipsilateral eye (beta = -9.17 and -12.77, respectively), as opposed to no opacification (P = <0.001 and <0.001, respectively). Eyes with grades 1,2 and 3 opacification of the sphenoid sinus also had a significantly less average GCIPL thickness than those with a clear sphenoid sinus (beta = -4.25, -5.28, and -7.60, P = 0.004, 0.003, and 0.003, respectively) (Table 4).

**Table 4:** Association between ophthalmic parameters and grade of specific sinus opacification in patients with chronic sinusitis.

		Mean Deviation (dB)		Pattern Standard Deviation (dB)		Average RNFL thickness (μm)		Average GCIPL thickness (μm)	
	Grade	Beta (95% CI)	P	Beta (95% CI)	P	Beta (95% CI)	P	Beta (95% CI)	P
Maxillary Sinus	0 (n = 29)	Reference		Reference		Reference		Reference	
	1 (n = 35)	0.49 (-0.85–1.83)	0.475	-0.07 (-0.49–0.35)	0.742	6.31 (-0.11–12.74)	0.054	-0.79 (-4.81–3.23)	0.701
	2 (n = 14)	0.29 (-1.43–2.01)	0.741	0.34 (-0.15–0.82)	0.172	-0.54 (-10.40–9.33)	0.915	-3.66 (-8.97–1.65)	0.177
	3 (n = 10)	1.84 (-0.77–4.46)	0.168	-1.05 (-1.83–-0.26)	**0.009**	9.16 (-1.32–19.63)	0.087	2.29 (-4.84–9.43)	0.529
Anterior Ethmoid Sinus	0 (n = 17)	Reference		Reference		Reference		Reference	
	1 (n = 38)	0.20 (-1.24–1.64)	0.785	0.47 (0.00–0.93)	**0.048**	-0.55 (-7.40–6.29)	0.874	1.33 (-2.62–5.29)	0.509
	2 (n = 19)	1.21 (0.68–3.09)	0.210	0.16 (-0.42–0.73)	0.593	0.57 (-7.71–8.85)	0.893	2.97 (-2.64–8.58)	0.299
	3 (n = 14)	-2.80 (-6.63–1.03)	0.151	0.60 (-0.15–1.35)	0.119	7.12 (-7.19–21.43)	0.329	3.40 (-9.40–16.20)	0.602
Posterior Ethmoid Sinus	0 (n = 43)	Reference		Reference		Reference		Reference	
	1 (n = 29)	0.30 (-1.22–1.81)	0.700	0.14 (-0.41–0.70)	0.613	2.62 (-2.39–7.62)	0.306	3.95 (0.39–7.86)	**0.048**
	2 (n = 8)	-2.96 (-5.10–-0.82)	**0.007**	1.68 (0.85–2.51)	**<0.001**	3.20 (-4.27–10.66)	0.402	0.96 (-2.95–4.88)	0.629
	3 (n = 8)	0.12 (-2.16–2.41)	0.917	-1.17 (-1.96–0.39)	**<0.003**	-0.02 (-10.89–10.86)	0.998	0.70 (-8.47–9.86)	0.882
Sphenoid Sinus	0 (n = 57)	Reference		Reference		Reference		Reference	
	1 (n = 18)	0.28 (-1.18–1.73)	0.708	0.04 (-0.38–0.47)	0.843	-12.77 (-18.79–-6.76)	**<0.001**	-4.25 (-7.17–-1.32)	**0.004**
	2 (n = 9)	-0.25 (-1.69–1.19)	0.735	1.29 (0.64–1.95)	**<0.001**	-9.17 (-14.01–-4.34)	**<0.001**	-5.28 (-8.77–-1.78)	**0.003**
	3 (n = 4)	-0.12 (-2.27–2.02)	0.910	0.14 (-0.25–0.53)	0.469	-12.87 (-21.70–-4.04)	**0.004**	-7.60 (-12.56–-2.64)	**0.003**
Frontal Sinus	0 (n = 56)	Reference		Reference		Reference		Reference	
	1 (n = 4)	-1.31 (-3.70–1.09)	0.285	0.59 (-0.36–1.54)	0.223	1.07 (-9.58–11.72)	0.844	-0.89 (-5.35–3.56)	0.695
	2 (n = 7)	0.93 (-0.34–2.20)	0.151	0.08 (-0.50–0.66)	0.794	-0.85 (-8.53–6.83)	0.829	-3.03 (-9.96–3.90)	0.392
	3 (n = 21)	0.57 (-0.81–1.95)	0.419	-0.41 (-0.98–0.17)	0.166	-7.93 (-17.38–1.53)	0.100	-4.58 (-11.43–2.28)	0.190
Osteomeatal Complex	0 (n = 39)	Reference		Reference		Reference		Reference	
	2 (n = 47)	-1.13 (-2.37–0.12)	0.075	0.12 (-0.45–0.68)	0.683	-1.15 (-7.33–5.04)	0.716		0.331

The severity of sinus inflammation was scored as 0 (no opacification), 1 (1–49% opacification), 2 (50–99% opacification), and 3 (total opacification); The osteomeatal complex was scored as either 0 (patent) or 2 (occluded).

Analyzed by generalized estimating equation adjusting adjusting age, sex, spherical equivalent, inter-eye correlation, and other sinus opacification.

Table 5 shows the ophthalmic parameters of the optic nerve based on the classification by Delano,[16] which is relates to the relationship between the sphenoid and posterior ethmoid sinuses and the optic nerve in eyes with ipsilateral chronic sinusitis. The frequencies of type 1, 2, 3, and 4 were 27 (52.9%), 11 (21.6%), 9 (17.6%), and 4 (7.8%), respectively, in chronic sinusitis patients. There was a significant difference in visual field (VF) mean deviation and pattern standard deviation among the groups (P < 0.001 and P = 0.006, respectively). In our post-hoc analysis, we found that the mean deviation in eyes with a type 4 relationship was significantly higher than that of eyes with types 1, 2, and 3 relationships (P <0.001, = 0.003, and = 0.006, respectively). The pattern standard deviation parameter was lower in type 4 and significantly different when compared to type 1 (P = 0.030) and type 2 (P = 0.002).

**Table 5:** Ophthalmic parameters among groups according to relationship between the sphenoid sinus, posterior ethmoid sinus, and the optic nerve in eyes with ipsilateral chronic sinusitis (n = 51).

	Type 1	Type 2	Type 3	Type 4	P
Frequency (n, %)	27 (52.9%)	11 (21.6%)	9 (17.6%)	4 (7.8%)	
VF					
Mean deviation	-1.41 ± 0.33 ^a^	-1.62 ± 0.87 ^a^	-2.86 ± 1.25 ^a^	0.84 ± 0.36 ^b^	**<0.001**
Pattern standard deviation	1.97 ± 0.22 ^a^	2.20 ± 0.32 ^a^	2.84 ± 0.71 ^a^^,^ ^b^	1.53 ± 0.26 ^b^	**0.006**
OCT RNFL parameters					
Average thickness	94.22 ± 2.31	97.56 ± 4.78	93.10 ± 6.00	98.08 ± 4.00	0.551
Temporal thickness	64.91 ± 1.50 ^a^	76.60 ± 3.48 ^b^	69.83 ± 2.36 ^b^	61.95 ± 1.60 ^a^	**<0.001**
Superior thickness	123.16 ± 3.92	121.33 ± 6.57	112.79 ± 10.08	128.27 ± 8.59	0.423
Nasal thickness	66.36 ± 1.73	68.97 ± 5.01	66.20 ± 2.63	70.77 ± 2.63	0.225
Inferior thickness	124.51 ± 3.95	122.42 ± 8.90	123.06 ± 10.47	131.14 ± 5.40	0.223
OCT GCIPL parameter (μm)					
Average thickness	81.35 ± 1.35 ^a^	82.05 ± 2.62 ^a^	82.18 ± 4.06 ^a^^,^ ^b^	89.41 ± 2.35 ^b^	**0.005**
Minimum thickness	78.20 ± 1.45	79.03 ± 2.24	80.30 ± 3.57	87.08 ± 3.10	0.052
Superotemporal thickness	80.64 ± 1.23	81.66 ± 2.80	83.14 ± 3.62	87.30 ± 3.05	0.209
Superior thickness	81.94 ± 1.53	83.42 ± 2.62	83.94 ± 4.47	88.20 ± 2.80	0.198
Superonasal thickness	83.51 ± 1.75 ^a^	82.75 ± 2.66 ^a^	84.91 ± 4.23 ^a^^,^ ^b^	91.52 ± 2.47 ^b^	**0.004**
Inferonasal thickness	81.03 ± 1.89 ^a^	80.58 ± 2.51 ^a^	83.77 ± 3.23 ^a^	91.68 ± 2.23 ^b^	**<0.001**
Inferior thickness	79.13 ± 1.34 ^a^	79.43 ± 2.80 ^a^	80.71 ± 3.55 ^a^	87.17 ± 2.33 ^b^	**0.005**
Inferotemporal thickness	81.34 ± 1.19 ^a^	84.07 ± 2.87 ^a^	85.60 ± 2.73 ^a^^,^ ^b^	89.85 ± 1.69 ^b^	**<0.001**

Type 1: ON lying adjacent to the superior and lateral walls of the sphenoid sinus.

Type 2: ON found to make an indentation on the sphenoid sinus.

Type 3: ON that traversed the sphenoid sinus.

Type 4: ON adjacent to the sphenoid and posterior ethmoid sinus and covered by aerated cells.

Data are presented as the mean ± SE.

^a, b^The same alphabetic superscript over the mean values indicates that the mean values are not statistically different from each other.

Analyzed by generalized estimating equation adjusting age, sex, spherical equivalent, and inter-eye correlation.

## Discussion

Chronic sinusitis is characterized by an inflammatory mucosal thickening and polyp formation in the paranasal sinuses.[2] Factors affecting chronic rhinosinusitis are very diverse, including genetic predisposition, immune disorders, mucociliary dysfunction, and structural abnormalities of the nasal cavity and paranasal sinuses. Along with these factors, if a bacterial infection in the sinuses are not adequately treated and become chronic, it progresses into chronic sinusitis. Such chronic inflammation may expand and cause compression of the surrounding structures or cause hypoxic conditions.[17] Thus, considering the positional relationship between the optic nerve and paranasal sinuses, various factors including inflammation and hypoxic conditions may have structural and functional effects on the adjacent optic nerve.

In this study, there was a positive correlation between the unilateral total Lund-Mackay score with the pattern standard deviation and no significant correlation with the mean deviation. Pattern standard deviation is known to have the limitation of not being appropriate when there is an advanced overall reduction in sensitivity, as a paradoxical decrease might occur.[18] We speculate that the pattern standard deviation may be a useful index, as all these patients have apparently mild optic neuropathies.

In this study, there was a negative linear relationship between the Lund-Mackay score, which represents the severity of inflammation in the paranasal sinuses, and the nasal RNFL thickness, which reflects the proximity of the optic nerve to the nasal side. This tendency may be attributed to the fact that the paranasal sinuses and orbital content are adjacent to each other, separated by a thin bony wall called the lamina papyracea, and the nasal side of the optic nerve is most likely to be affected by inflammation of the sinus. Nasal RNFL thickness can be affected by refractive error and usually measures thinner in myopia.[19] However, there was no difference in refractive error between the control and chronic sinusitis groups in the study population (P = 0.290) and no correlation was found between spherical equivalent and Lund-Mackay score in chronic sinusitis eyes (P = 0.848). In addition, all statistical analysis in this study were performed adjusting refractive error. We speculate that the apparent normality of the vertical axis RNFL might be due to the persistent inflammation of optic nerve which induces RNFL edema.

In the analysis between the Lund-Mackay score and OCT GCIPL parameter, the average, minimum, superotemporal, superior, superonasal, and inferonasal GCIPL thickness were negatively correlated with the score (all, P < 0.05). Studies in the neuro-ophthalmological field have shown that the GCIPL index reflects better the optic nerve changes than RNFL in multiple sclerosis-related optic neuritis[20] and optic neuritis.[21–23] GCIPL thickness probably represents a more accurate parameter of axonal loss, and this concept corresponds to our results.

We found that chronic inflammation of the sphenoid sinus can affect the thickness of the optic nerve. The sphenoid sinus is intimately related to the carotid artery, the optic nerve, and the vidian nerve. These structures, which are present before sinus development, produce irregularities in the walls of the sinus as the cavity develops. In well-pneumatized cavities, only a thin bony plate separates the sinus from adjacent structures.[7] There have been some sporadic case reports on the inflammation of the sphenoid sinus causing optic neuropathy.[10, 24] Our study is meaningful in that it is the first to demonstrate the association between sphenoid sinusitis and optic nerve change.

The posterior ethmoid sinus and optic nerve are also anatomically close. This is especially true in patients with a highly pneumatized posterior ethmoid sinus (Onodi cell). When acute inflammation of an Onodi cell occurs, it affects vision through direct compression by a mucocele or through the spread of inflammation. In this study, patients with grade 2 inflammation of the posterior ethmoid sinus showed a lower mean deviation and higher pattern standard deviation compared to those with a clear sinus. However, patients with other grades of inflammation did not show the tendency. Moreover, patients with grade 3 inflammation of the posterior ethmoid sinus showed a lower pattern standard deviation compared to those with a clear sinus. This is probably due to the insufficient sample size in each group. Another explanation is that fewer patients with better posterior ethmoid sinus pneumatization were present in our patient group.

Researchers who have analyzed the correlation between the sphenoid sinus and optic nerve suggest four types. In Type 1, the optic nerve is only adjacent to the superolateral wall of the sphenoid sinus. However, in Types 2 and 3, the nerve begins to protrude into the sinus, and in Type 4, it is completely exposed to the inside of the sinus, surrounded by aerated cells.^17^ This anatomic variation is important to a rhinologic surgeon performing sinus surgery. Also in patients with an exposed optic nerve, acute inflammatory sinusitis or rapid expansion of sinus lesions such as a mucocele may cause acute, complete unilateral blindness. In addition to these dangers, bony dehiscence of the optic nerve protruding into the sphenoid sinus causes more vulnerability to infection, inflammation, and compression hypoxia. To the best of our knowledge based on a literature review, our study was the first to investigate the effect of chronic inflammation on the optic nerve based on this anatomical relationship.

**Fig 4:**
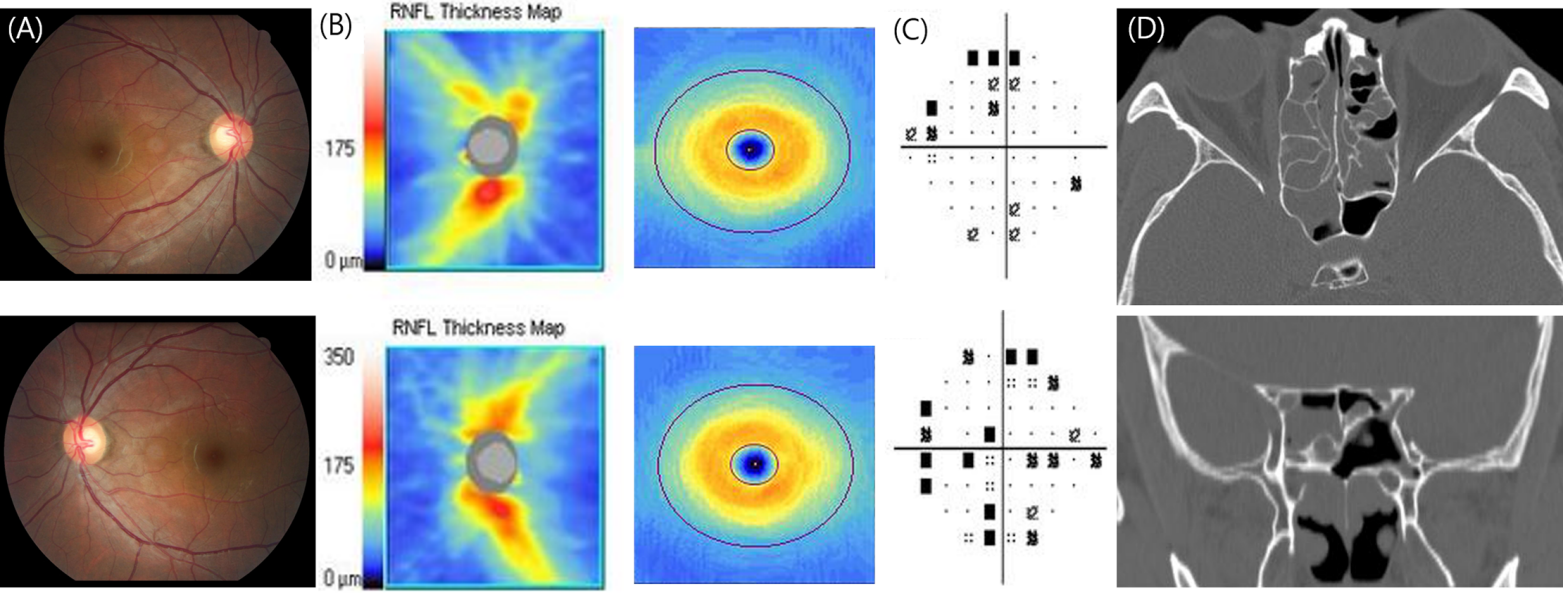
Case. (A) The optic discs appear normal on fundus photography (upper, right eye; lower, left eye). (B) On the OCT RNFL map, there is subtle thinning of the superior aspect of the retinal nerve fiber layer in the right eye (upper, right eye; lower, left eye). OCT GCIPL map shows normal finding. (C) Reliable automated perimetry demonstrates generalized decreased sensitivity in both eyes (upper, right eye; lower, left eye) (mean deviation -4.62 dB, pattern standard deviation 5.40 dB in his right eye; mean deviation -7.85 dB, pattern standard deviation 5.68 dB in his left eye). (D) The ostiomeatal CT image shows sinusitis of the posterior ethmoid and sphenoid sinuses. In addition, the optic nerve protrudes into a well pneumatized posterior ethmoid sinus (Onodi cell) with some bony dehiscence.

Fig 4 shows the case of a 33-year-old man with chronic sinusitis and a Type 3 relationship between the paranasal sinuses and optic nerve. In this patient, the posterior ethmoid sinus was well pneumatized. In this case, the pneumatization of the posterior ethmoid sinus extended more posteriorly and was located superior to the sphenoid sinus. This is known as an “Onodi cell”. As the optic nerve protrudes into an Onodi cell, it is more vulnerable to damage caused by intraoperative complications and/or acute inflammation. The anatomical feature where the optic nerve passes through the sphenoid sinus might affect the vulnerability of optic nerve in the referred case. Since only 9 eyes with a Type 3 relationship and only 4 eyes with a Type 4 relationship were included in our study, further study with a sufficient sample size in each group is warranted in order to investigate the susceptible anatomic features that lead to structural and functional optic nerve damage.

The findings in this study would be stronger if both functional and structural parameters correlated with severity score at the same time. However, quantification of visual function is on a nonlinear decibel scale (dB), whereas retinal structural parameter is on a linear scale (μm). The units of measurement for structural and functional parameters could be a confounding factor. We could not investigate a correlation between duration of opacity and ophthalmic change. Our patient group was chronic sinusitis patients who did not respond to medication for more than 3 months. However, accurate information about the duration of the patient 's illness could not be obtained. We did not include color vision evaluation which is frequently abnormal inflammatory optic neuropathies for optic nerve function analysis. The clinical significance of the finding that asymptomatic optic nerve changes occur in chronic sinusitis cannot be emphasized because most groups of chronic sinusitis had OCT and VF results within normal limits.

This study was the first to confirm the effect of chronic sinusitis on the optic nerve. The ophthalmic parameter most affected by the severity of chronic sinusitis was the VF pattern standard deviation, OCT nasal RNFL thickness, and OCT average, minimum, superotemporal, superior, and superonasal, thickness. Inflammation of the posterior ethmoid and sphenoid sinus was associated with optic nerve changes to a greater extent than that of the other paranasal sinuses. Chronic sinusitis can adversely affect the optic nerve, so sinusitis should be treated properly. In addition, the ophthalmologist should consider the possibility of structural functional changes when evaluating optic nerve in patients with sinusitis. Considering the diversity of the anatomical feature where the optic nerve passes through the sphenoid and ethmoid sinuses and small sample size of the study, longitudinal studies investigating the relationship between chronic sinusitis and optic nerve damage are warranted in future.
